# The levels of circulating cytokines and risk of neuromyelitis optica spectrum disorder: a Mendelian randomization study

**DOI:** 10.3389/fimmu.2024.1418309

**Published:** 2024-07-01

**Authors:** Xue Ma, Yao Wang, Xin Chen, Jun Guo

**Affiliations:** ^1^ Department of Neurology, Tangdu Hospital, Air Force Medical University, Xi’an, China; ^2^ Department of Neurology, The First Affiliated Hospital of Xi’an Jiao Tong University, Xi’an, China

**Keywords:** neuromyelitis optica spectrum disorder, cytokine, chemokine, inflammation, Mendelian randomization

## Abstract

**Background:**

Neuromyelitis optica spectrum disorder (NMOSD) is an inflammatory autoimmune disease affecting the central nervous system (CNS). NMOSD pathogenesis involves systemic inflammation. However, a causal relationship between circulating cytokine levels and NMOSD remains unclear.

**Methods:**

Mendelian randomization (MR) approaches were used to investigate the potential association between genetically determined circulating 19 inflammatory cytokines and 12 chemokines levels and the risk of developing NMOSD.

**Results:**

After Bonferroni correction, the risk of aquaporin 4-antibody (AQP4-ab)-positive NMOSD was suggested to be causally associated with the circulating levels of three cytokines, including interleukin (IL)-4 [odds ratio (OR): 11.01, 95% confidence interval (CI): 1.16–104.56, *P* = 0.037], IL-24 (OR: 161.37; 95% CI: 2.46–10569.21, *P* = 0.017), and C-C motif chemokine 19 (CCL19) (OR: 6.87, 95% CI: 1.78–26.93, *P* = 0.006).

**Conclusion:**

These findings suggest that a genetic predisposition to higher levels of IL-4, IL-24, and CCL19 may exert a causal effect on the risk of AQP4-ab-positive NMOSD. Further studies are warranted to clarify how these cytokines affect the development of AQP4-ab-positive NMOSD.

## 1 Introduction

Neuromyelitis optica spectrum disorder (NMOSD) is a chronic autoimmune inflammatory disease that affects the central nervous system (CNS) and is characterized by recurrent episodes of optic neuritis and transverse myelitis (1). Serum aquaporin 4-antibody (AQP4-ab) is positive in more than 80% of patients with NMOSD, referred to as AQP4-ab-positive NMOSD (2). Patients without AQP4-ab are considered AQP4-ab-negative NMOSD (3).

The etiology of NMOSD remains elusive. Multiple risk factors, including human leukocyte antigen genetic predisposition and infection, may play pivotal roles in susceptibility to NMOSD (4–6). Numerous studies have suggested that systemic inflammatory factors, such as elevated cytokine and chemokine levels, may participate in CNS demyelinating lesions in NMOSD and may serve as therapeutic targets (7, 8). Cytokines are low-molecular weight proteins that regulate immune and inflammatory responses. Cytokines have been found to play a vital role in the regulation of neuroinflammatory responses by recruiting and activating different cell types (9). A review reported higher serum concentrations of interleukin-4 (IL-4), IL-6, IL-17A, IL-21, IL-23, IL-32, interferon-gamma (IFN-γ), and tumor necrosis factor-alpha (TNF-α) in patients with NMOSD than in controls (7). Elevated levels of serum chemokines, such as monocyte chemoattractant protein-1 and monocyte chemoattractant protein-4, were observed in patients with NMOSD (10). Furthermore, IL-6 receptor (IL-6R)-blocking therapy has demonstrated efficacy in reducing the annualized relapse rate in patients with NMOSD (11). IFN-γ and IL-17A are potential therapeutic targets for NMOSD (12). However, the associations between the levels of several cytokines and the risk of NMOSD remain elusive.

Mendelian randomization (MR) analysis is a useful statistical framework for assessing causality between exposures and outcomes by utilizing genetic variants as instrumental variables (IVs) (13). Considering that genetic variants are fixed and allocated randomly during conception and that alleles are not influenced by environmental or lifestyle factors, this method can minimize the effects of confounding factors and reverse causation (13). Thus, MR may offer more robust evidence of causal effects (14). Recently, a genome-wide association study (GWAS) meta-analysis evaluated the genetic basis of multiple circulating cytokines (15), which provides an opportunity to investigate their correlations with NMOSD. Using a two-sample MR method, we comprehensively assessed the plausible causal relationships between circulating cytokine levels and susceptibility to NMOSD.

## 2 Methods

### 2.1 Data source and study design

This MR study was based on publicly available GWAS databases and no additional ethical approval was needed.

Detailed information on the exposure and outcome traits are presented in **Supplementary Table 1**. Nineteen inflammatory cytokines and 12 chemokines were chosen as exposures from the largest and most recent GWAS comprising 14, 828 European individuals to date (15). Summary-level data for NMOSD patients were extracted from a shared dataset (6). A total of 215 patients with NMOSD were recruited from the European population, including 132 AQP4-ab-positive patients, 83 AQP4-ab-negative patients, and 1244 healthy participants. These datasets are available upon request.

### 2.2 Instrument variable selection

**Figure 1:**
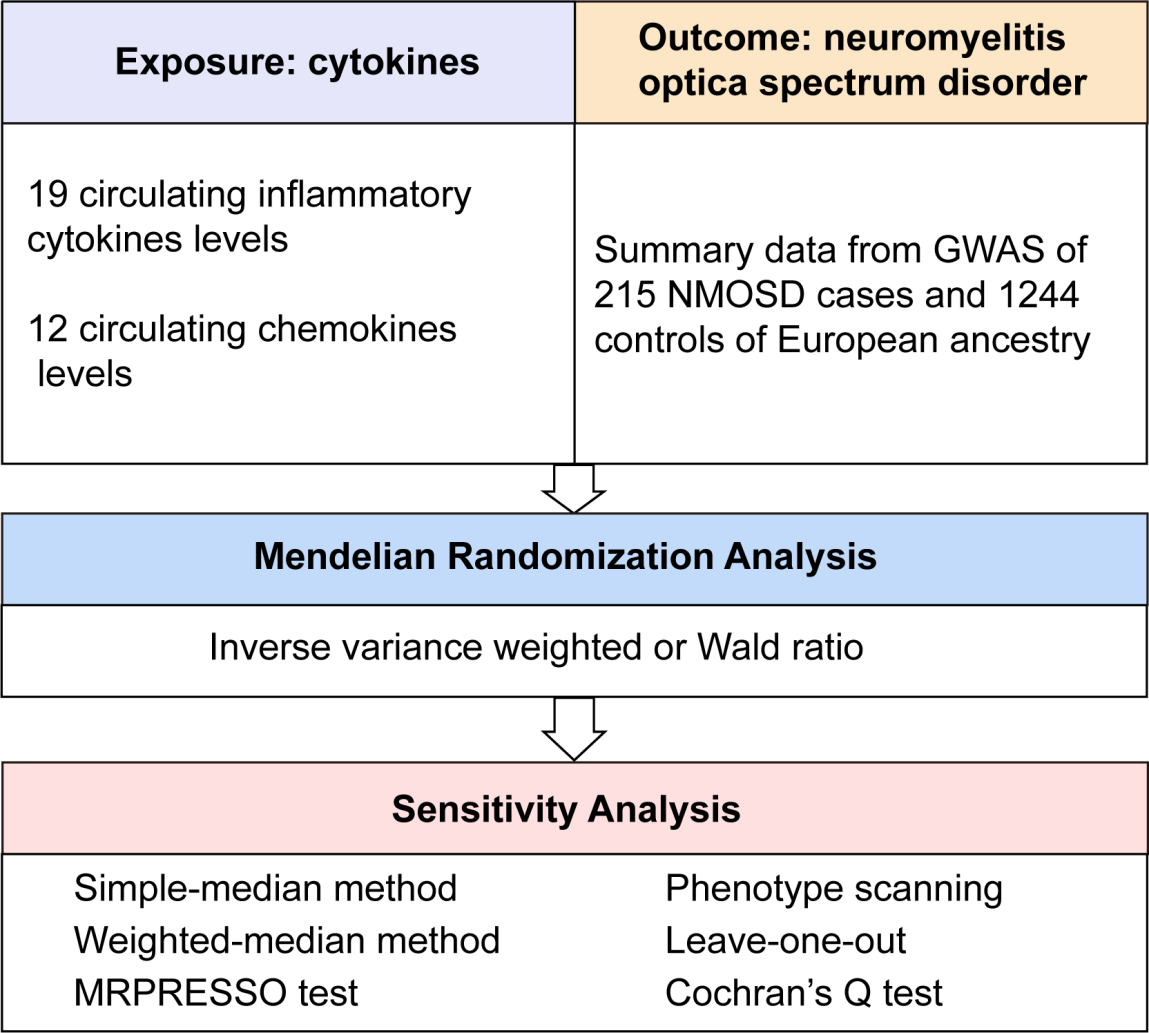
Overall design of the present study. GWAS, genome-wide association study; MRPRESSO, MR pleiotropy residual sum and outlier test; NMOSD, neuromyelitis optica spectrum disorder.

First, the relevance assumption was satisfied, as all single nucleotide polymorphisms (SNPs) achieved genome-wide significance. A lenient significance threshold (*P* < 5× 10^−6^) was adopted to select instrumental variables (IVs). Second, the independence assumption was confirmed, as IVs exhibited no correlation with other confounding factors. Third, in addition to exposure factors, IVs did not influence outcomes through alternative pathways (16). The graphical concept of the MR design is shown in **Figure 1**.

### 2.3 Statistical analyses

The selected genetic variants as instruments were strongly associated with exposure (*P* < 5 × 10^−6^) and independent [linkage disequilibrium (LD) r^2^ < 0.001], and the datasets were harmonized in accordance with a prior methodology (17). The F-statistic was computed to assess the robustness of the IVs (F-statistic > 10) (18). The variation explained by the individual genetic instrument was R^2^, which was calculated using the formula ${\text{R}}^{2}\text{= (beta}\times {\sqrt{2\times MAF\left(1-MAF\right))}}^{2}$ . The effect of the genetic variant on each cytokine is beta, and the minor allele frequency is the MAF (19). Depending on the number of selected genetic instruments in the primary MR analysis, the Wald ratio or inverse-variance weighted fixed methods were used to estimate causal effects.

Sensitivity analyses were performed to ensure the robustness of the primary MR findings. We removed from the initial set of instruments for which we found biological or statistical evidence of pleiotropy. Potential directional pleiotropy was evaluated using the MR-Egger regression intercept and MRPRESSO global test (20, 21). Leave-one-out (LOO) was conducted to detect horizontal pleiotropy (22). Funnel and scatter plots were constructed to visually inspect symmetry and effect estimates. In addition, we used Cochrane Q statistics to evaluate heterogeneity, and a *P*-value < 0.05 was considered to indicate heterogeneity.

Bonferroni correction was employed to correct for multiple testing and to establish statistical significance at a *P*-value < 1.56 × 10^−3^ (0.05/31), considering the number of cytokines. *P*-values within the range of 1.56 × 10^−3^ to 0.05 were interpreted as indicative of potential causal associations (23). All the statistical analyses were performed using R software (v.4.1.1). MR analyses were conducted using the TwoSampleMR (v.0.5.6) and Mendelian randomization (v.0.5.1) R packages (24, 25).

## 3 Results

First, for AQP4-ab-positive NMOSD, SNPs with P < 5 × 10−8 were selected and clumped at LD r2 = 0.001, and nine cytokines, including IL-6, IL-8, IL-10, IL-12β, IL-18, CCL4, CCL19, CXCL5, CXCL6, and CXCL9, were selected for more than one SNP as IVs (**Supplementary Table 2**). In addition, only two cytokines (IL-6 and IL-12β) included more than three SNPs for AQP4-ab-positive NMOSD. For AQP4-ab-negative NMOSD, at the P < 5 × 10−8 threshold, nine cytokines, including IL-6, IL-8, IL-10, IL-12β, IL-18, CCL19, CXCL5, CXCL6, and CXCL9, had more than one SNP as an IV, and three cytokines (IL-6, IL-12β, and CXCL5) had more than three SNPs as IVs (**Supplementary Table 3**). Considering the limited number of SNPs, a liberalizing threshold of a P-value of 5 × 10−6 was adopted to select IVs.

**Figure 2:**
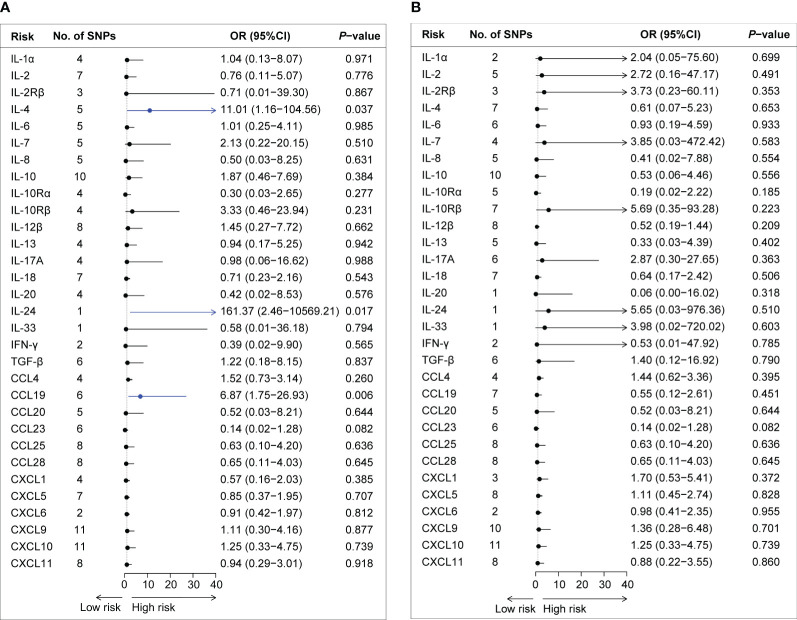
Forest plot of the Mendelian randomization analyses for the associations between circulating cytokine levels and the risk of neuromyelitis optica spectrum disorder. Forest plot of the MR results for the relationships between circulating cytokines and the risk of AQP4-ab-positive NMOSD **(A)** and AQP4-ab-negative NMOSD **(B)**. No., number; OR, odds ratio; CI, confidence interval; IL-1α, interleukin-1α; IL-2, interleukin-2; IL-2Rβ, interleukin-2 receptor β; IL-4, interleukin-4; IL-6, interleukin-6; IL-7, interleukin-7; IL-8. interleukin-8; IL-10, interleukin-10; IL-10Rα, interleukin-10 receptor α; IL-10Rβ, interleukin-10 receptor β; IL-12β, interleukin-12β; IL-13, interleukin-13; IL-17A, interleukin-17A; IL-18, interleukin-18; IL-20, interleukin-20; IL-24, interleukin-24; IL-33, interleukin-33; IFN-γ, interferon-gamma; TGF-β, transforming growth factor β; CCL4, C-C motif chemokine 4; CCL19, C-C motif chemokine 19; CCL20, C-C motif chemokine 20; CCL23, C-C motif chemokine 23; CCL25, C-C motif chemokine 25; CCL28, C-C motif chemokine 28; CXCL1, C-X-C motif chemokine 1; CXCL5, C-X-C motif chemokine 5; CXCL6, C-X-C motif chemokine 6; CXCL9, C-X-C motif chemokine 9; CXCL10, C-X-C motif chemokine 10; CXCL11, C-X-C motif chemokine 11; SNPs, single nucleotide polymorphisms.

By applying these selection criteria (r2 < 0.001, P < 5 × 10−6), we identified 338 SNPs associated with 31 cytokines for AQP4-ab-positive and AQP4-ab-negative NMOSD patients. The F-statistic of individual variants ranged from 20.83 to 1010.97 for AQP4-ab-positive NMOSD patients and from 20.84 to 1010.97 for AQP4-ab-negative NMOSD patients. Instruments for each cytokine explained the proportional variance from 0.2% to 7.9% for AQP4-ab-positive NMOSD, and from 0.1% to 7.6% for AQP4-ab-negative NMOSD. The results of the IVW method regarding the associations between the 31 cytokines and AQP4-ab-positive and AQP4-ab-negative NMOSD patients are illustrated in **Figures 2A, B**. Following Bonferroni correction, only two inflammatory cytokines (IL-4 and IL-24) and one chemokine (CCL19) exhibited suggestive associations with the risk of AQP4-ab-positive NMOSD. Summary data for the genetic variants associated with IL-4, IL-24, and CCL19 are presented in **Supplementary Table 4**.

The IVW method provided suggestive evidence that an elevated IL-4 level was associated with an increased risk of NMOSD [odds ratio (OR): 11.01, 95% confidence interval (CI): 1.16–104.56, P = 0.037]. Furthermore, our findings suggested an association between a genetically determined higher circulating level of IL-24 and an increased risk of AQP4-ab-NMOSD using the Wald ratio method (OR: 1.10, 95% CI: 1.03–1.17, P = 0.005). Sensitivity analysis and the GWAS Catalog did not indicate documented pleiotropy of genetic variants.

Among the 12 chemokines, we observed suggestive evidence that circulating levels of CCL19 were positively associated with AQP4-ab-positive NMOSD risk (OR: 6.87, 95% CI: 1.75–26.93, P = 0.006). Sensitivity analyses revealed consistent trends (OR: 15.45, 95% CI: 1.20–199.40, P = 0.099 by simple mode method; OR: 6.68, 95% CI: 1.15–38.71, P = 0.034 by weighted median method). No heterogeneity was observed according to Cochran’s Q test. No evidence of SNPs disproportionally affecting MR estimates was detected by single-SNP or LOO analysis (data not shown).

## 4 Discussion

A potential connection between cytokines and the development of NMOSD has been suggested by previous observational studies. However, the influence of confounding factors and reverse causation can undermine reliability of these observational studies. In this study, we assessed a possible causal relationship between circulating levels of 31 cytokines and the risk of NMOSD using an MR design, which is a method that probe potential causality on the effect of exposures on outcomes by leveraging genetic information. Our findings provide suggestive evidence that genetically predicted circulating levels of T helper 2 cell (Th2)-type inflammatory cytokines (IL-4 and IL-24) and CCL19 are linked to susceptibility to AQP4-ab-positive NMOSD, revealing the involvement of the Th2 response in the development of AQP4-ab-positive NMOSD.

Th2 predominance may be involved in the pathogenesis of NMOSD (26). IL-4, a pleiotropic cytokine, is responsible for the polarization of Th2 (27). IL-4 also serves as a B-cell stimulating factor that induces the differentiation of B cells (28). In asthmatic animals, IL-4 leads to a systemic inflammatory response in peripheral blood neutrophils by increasing the production of proinflammatory IL-8 and TNF-α (29). Anti-IL-4 monoclonal antibodies reduce the severity of experimental autoimmune myocarditis (30). In contrast, IL-4 exerts an anti-inflammatory effect that suppresses the development multiple sclerosis in mouse models (31, 32). These data suggest that IL-4 may play a dual role in autoimmune diseases. Elevated serum IL-4 levels were observed in patients with NMOSD (33). Further comprehensive experimental studies are needed to elucidate the role of IL-4 in NMOSD.

IL-24 belongs to the IL-20 subfamily of cytokines and is a member of the IL-10 cytokine family (34). IL-24 is primarily produced by Th2 cells and is classified as a Th2-type cytokine (35). Transcription factors, such as signal transducer and activator of transcription 6 STAT6 and GATA-binding protein 3, are presumed to regulate IL-24 expression (36). IL-24 can regulate various types of immune cells, including T cells (37), B cells (38), and natural killer cells (39). Increased levels of IL-24 are associated with chronic autoimmune diseases, such as psoriasis (40), rheumatoid arthritis (41), and inflammatory bowel disease (42). However, the role of IL-24 in NMOSD has not been reported.

The chemokine CCL19, alternatively referred to as macrophage inflammatory protein-3 or EBV-induced molecule 1 ligand, is predominantly synthesized in lymphoid tissues (43, 44). The receptor of CCL19 is C-C motif chemokine receptor 7, which is expressed on mature dendritic cells, B cells, naïve T cells, and central memory T cells (45). In allergic diseases, CCL19 facilitates Th2 differentiation and contributes to allergic airway inflammation (46). A previous study demonstrated elevated CCL19 levels in the cerebrospinal fluid of patients with NMOSD during the relapse phase (47). Nevertheless, further investigation is warranted to elucidate the role of CCL19 in NMOSD.

However, this study had several limitations. First, all the participants included in our study were of European ancestry, which may limit the generalizability of our findings to other racial groups. Second, a relaxed significance threshold of P < 5 × 10−6 was applied to select IVs, which may cause false-positive variants and bias. However, the F-statistics of the IVs were all > 10, indicating a reduced probability of weak instrument bias. Similarly, the same significance threshold (P < 5 × 10−6) was adopted by several other studies that explored the correlations between cytokine levels and Alzheimer’s disease (48), ALS (49), and cognitive decline (50). Third, although we attempted to identify potential secondary phenotypes of IVs using the GWAS catalog, pleiotropy cannot be completely ruled out. Fourth, MR results indicated that none of the cytokines exhibited a statistically significant association with the risk of NMOSD. After Bonferroni correction, only three of these cytokines (IL-4, IL-24, and CCL19) showed suggestive associations. Moreover, we were only able to access the GWAS dataset for NMOSD, which included 215 patients with NMOSD, consisting of 132 AQP4-ab-positive patients and 83 AQP4-ab-negative patients. Given the low incidence of NMOSD in European countries, the number of enrolled patients was relatively low. As a result, the potential association between these cytokines and NMOSD risk requires further validation using GWAS datasets with larger cohorts. Finally, although these included cytokines may not directly contribute to the risk of NMOSD, they can affect disease progression or survival. For instance, inhibition of IL-6R activity effectively prevents NMOSD relapse (11). However, this association was not addressed in our MR analysis. Therefore, further studies are needed to ascertain whether cytokines play a role in exacerbating or ameliorating NMOSD.

## 5 Conclusion

Our MR study supported the suggestive causal associations between the circulating levels of three cytokines (IL-4, IL-24, and CCL19) and an increased risk of AQP4-ab-positive NMOSD. Further researches are necessary to validate these findings and determine whether they could be potential therapeutic targets.

## Data availability statement

The datasets presented in this study can be found in online repositories. The names of the repository/repositories and accession number(s) can be found in the article/**Supplementary Material**.

## Ethics statement

This MR study was based on publicly available GWAS databases and no additional ethical approval was needed. The studies were conducted in accordance with the local legislation and institutional requirements.

## Author contributions

XM: Conceptualization, Funding acquisition, Supervision, Writing – original draft, Writing – review & editing. YW: Conceptualization, Formal analysis, Writing – original draft, Writing – review & editing. XC: Conceptualization, Data curation, Methodology, Writing – original draft, Writing – review & editing. JG: Formal analysis, Funding acquisition, Supervision, Writing – review & editing, Writing – original draft.
