# Supplementary material for: The levels of circulating cytokines and risk of neuromyelitis optica spectrum disorder: a Mendelian randomization study
Source: Front Immunol. 2024 Jul 1;15:1418309. doi: 10.3389/fimmu.2024.1418309 (PMC11246864; doi:10.3389/fimmu.2024.1418309)
Supplement: Supplementary file 1 [file Table_1.docx]

Supplementary Material

# Supplementary Tables

**1.1 Supplementary Table 1. Description of the data sources**

| Trait | References | Sample size | Phenotype ascertainment |
| --- | --- | --- | --- |
| Exposure | | | |
| Cytokines | Zhao JH, et al.^1^ 2023 | 2935 | Immunoassay techniques |
| Outcome | | | |
| NMOSD | Estrada, et al.^2^ 2018 | 215 patients with NMOSD, including 132 AQP4-ab-positive patients and 83 AQP4-ab-negative patients, and 1244 normal controls | The 2006 NMO diagnostic criteria |

Abbreviations: AQP4-ab, aquaporin 4-antibody; NMOSD, neuromyelitis optica spectrum disorder.

**1.2 Supplementary Table 2. Details of the number of genetic instruments and F-statistic for each cytokine for AQP4-ab-positive NMOSD**

| Cytokines | Abbreviations | *P* < 5×10^-8^ | | | *P* < 5×10^-6^ | | |
| --- | --- | --- | --- | --- | --- | --- | --- |
|  |  | No. of SNPs | *F*-statistic (range) | Overall R^2^ | No. of SNPs | F-statistic (range) | Overall R^2^ |
| Interleukin-1α | IL-1α | 0 | / | / | 4 | 21.56 (20.93-22.27) | 0.006 |
| Interleukin-2 | IL-2 | 0 | / | / | 7 | 22.53 (20.85-24.94) | 0.015 |
| Interleukin-2 receptor β | IL-2Rβ | 0 | / | / | 3 | 23.28 (22.65-23.88) | 0.005 |
| Interleukin-4 | IL-4 | 0 | / | / | 5 | 22.17 (20.95-24.79) | 0.010 |
| Interleukin-6 | IL-6 | 5 | 57.12 (20.97-197.55) | 0.020 | 5 | 57.12 (20.97-197.55) | 0.020 |
| Interleukin-7 | IL-7 | 0 | / | / | 5 | 23.97 (22.35-25.02) | 0.009 |
| Interleukin-8 | IL-8 | 2 | 47.24 (32.98-61.50) | 0.006 | 5 | 31.11 (21.06-61.50) | 0.013 |
| Interleukin-10 | IL-10 | 2 | 39.23 (35.09-43.37) | 0.005 | 10 | 25.31 (21.05-43.37) | 0.020 |
| Interleukin-10 receptor α | IL-10Rα | 0 | / | / | 4 | 22.60 (20.95-24.44) | 0.006 |
| Interleukin-10 receptor β | IL-10Rβ | 0 | / | / | 4 | 21.63 (21.17-22.13) | 0.009 |
| Interleukin-12 subunit beta | IL-12β | 5 | 131.13 (31.79-468.95) | 0.044 | 8 | 91.50 (21.93-468.95) | 0.049 |
| Interleukin-13 | IL-13 | 0 | / | / | 4 | 24.91(21.61-31.27) | 0.008 |
| Interleukin-17A | IL-17A | 0 | / | / | 4 | 22.95 (20.86-24.91) | 0.008 |
| Interleukin-18 | IL-18 | 3 | 98.55 (35.38-224.45) | 0.020 | 7 | 48.85 (21.01-224.45) | 0.030 |
| Interleukin-20 | IL-20 | 0 | / | / | 4 | 22.09 (20.94-24.49) | 0.006 |
| Interleukin-24 | IL-24 | 0 | / | / | 1 | 23.29 | 0.002 |
| Interleukin-33 | IL-33 | 0 | / | / | 1 | 21.41 | 0.004 |
| Interferon-γ | IFN-γ | 0 | / | / | 2 | 21.99 (21.38-22.60) | 0.003 |
| Transforming growth factor β | TGF-β | 0 | / | / | 6 | 23.24 (21.49-26.71) | 0.013 |
| C-C motif chemokine 4 | CCL4 | 2 | 457.60 (30.48-884.72) | 0.064 | 4 | 196.47 (20.89-886.68) | 0.066 |
| C-C motif chemokine 19 | CCL19 | 1 | 168.65 (42.48-304.41) | 0.034 | 6 | 62.85 (21.05-304.45) | 0.047 |
| C-C motif chemokine 20 | CCL20 | 0 | / | / | 5 | 38.19 (20.96-61.93) | 0.013 |
| C-C motif chemokine 23 | CCL23 | 0 | / | / | 6 | 22.33 (20.83-25.31) | 0.013 |
| C-C motif chemokine 25 | CCL25 | 0 | / | / | 8 | 45.17 (21.95-219.36) | 0.032 |
| C-C motif chemokine 28 | CCL28 | 0 | / | / | 8 | 24.62 (20.92-32.96) | 0.017 |
| C-X-C motif chemokine 1 | CXCL1 | 0 | / | / | 4 | 124.25 (20.85-527.46) | 0.042 |
| C-X-C motif chemokine 5 | CXCL5 | 3 | 218.54 (31.35-742.89) | 0.060 | 7 | 110.29 (21.05-743.12) | 0.067 |
| C-X-C motif chemokine 6 | CXCL6 | 2 | 377.07 (50.17-1008.42) | 0.079 | 2 | 377.92 (50.17-1010.97) | 0.079 |
| C-X-C motif chemokine 9 | CXCL9 | 3 | 69.05 (31.17-121.86) | 0.014 | 11 | 35.61 (20.90-121.87) | 0.026 |
| C-X-C motif chemokine 10 | CXCL10 | 0 | / | / | 11 | 37.93 (21.17-150.88) | 0.029 |
| C-X-C motif chemokine 11 | CXCL11 | 0 | / | / | 8 | 44.38 (21.14-210.40) | 0.027 |

Abbreviations: No., number; SNPs, single nucleotide polymorphisms.

**1.3 Supplementary Table 3. Details of the number of genetic instruments and F-statistic for each cytokine for AQP4-ab-negative NMOSD.**

| Cytokines | Abbreviations | *P* < 5×10^-8^ | | | *P* < 5×10^-6^ | | |
| --- | --- | --- | --- | --- | --- | --- | --- |
|  |  | No. of SNPs | *F*-statistic (range) | Overall R^2^ | No. of SNPs | F-statistic (range) | Overall R^2^ |
| Interleukin-1α | IL-1α | 0 | / | / | 2 | 20.94 (20.93-20.95) | 0.003 |
| Interleukin-2 | IL-2 | 0 | / | / | 5 | 22.81 (21.22-24.94) | 0.012 |
| Interleukin-2 receptor β | IL-2Rβ | 0 | / | / | 3 | 22.84 (21.32-23.88) | 0.005 |
| Interleukin-4 | IL-4 | 0 | / | / | 7 | 22.51 (20.96-24.81) | 0.011 |
| Interleukin-6 | IL-6 | 4 | 23.56 (21.12-27.02) | 0.008 | 6 | 51.41 (20.97-197.54) | 0.021 |
| Interleukin-7 | IL-7 | 0 | / | / | 4 | 24.38 (23.91-25.02) | 0.007 |
| Interleukin-8 | IL-8 | 2 | 47.24 (32.98-61.50) | 0.006 | 5 | 31.11 (21.06-61.50) | 0.013 |
| Interleukin-10 | IL-10 | 2 | 39.23 (35.09-43.37) | 0.005 | 10 | 25.47 (21.05-43.37) | 0.021 |
| Interleukin-10 receptor α | IL-10Rα | 0 | / | / | 5 | 22.48 (20.95-24.44) | 0.008 |
| Interleukin-10 receptor β | IL-10Rβ | 0 | / | / | 7 | 22.60 (21.17-25.91) | 0.011 |
| Interleukin-12 subunit beta | IL-12β | 4 | 155.97 (42.67-468.95) | 0.042 | 8 | 90.21 (21.49-468.95) | 0.048 |
| Interleukin-13 | IL-13 | 0 | / | / | 5 | 24.91 (21.61-31.27) | 0.008 |
| Interleukin-17A | IL-17A | 0 | / | / | 6 | 23.22 (20.86-27.54) | 0.013 |
| Interleukin-18 | IL-18 | 3 | 98.55 (35.38-224.45) | 0.020 | 7 | 48.77 (21.02-224.45) | 0.030 |
| Interleukin-20 | IL-20 | 0 | / | / | 1 | 21.45 | 0.001 |
| Interleukin-24 | IL-24 | 0 | / | / | 1 | 23.30 | 0.002 |
| Interleukin-33 | IL-33 | 0 | / | / | 1 | 21.85 | 0.004 |
| Interferon-γ | IFN-γ | 0 | / | / | 2 | 22.00 (21.39-22.61) | 0.003 |
| Transforming growth factor β | TGF-β | 0 | / | / | 6 | 23.24 (21.49-26.71) | 0.013 |
| C-C motif chemokine 4 | CCL4 | 1 | 886.68 | 0.060 | 4 | 165.74 (20.85-886.67) | 0.067 |
| C-C motif chemokine 19 | CCL19 | 2 | 144.95 (42.49-  304.4526) | 0.039 | 7 | 63.64 (21.05-304.45) | 0.052 |
| C-C motif chemokine 20 | CCL20 | 0 | / | / | 5 | 38.20 (20.95-61.93) | 0.013 |
| C-C motif chemokine 23 | CCL23 | 0 | / | / | 6 | 22.33 (20.84-25.31) | 0.012 |
| C-C motif chemokine 25 | CCL25 | 0 | / | / | 8 | 45.27 (21.95- 220.20) | 0.028 |
| C-C motif chemokine 28 | CCL28 | 0 | / | / | 8 | 24.63 (20.92-32.96) | 0.017 |
| C-X-C motif chemokine 1 | CXCL1 | 0 | / | / | 3 | 150.10 (21.05-527.46) | 0.041 |
| C-X-C motif chemokine 5 | CXCL5 | 4 | 218.54 (31.35-743.12) | 0.063 | 8 | 104.67 (21.05-743.12) | 0.071 |
| C-X-C motif chemokine 6 | CXCL6 | 3 | 377.92 (50.17-1010.97) | 0.076 | 2 | 377.92 (50.17-1010.97) | 0.076 |
| C-X-C motif chemokine 9 | CXCL9 | 3 | 69.05 (31.18-121.87) | 0.0140 | 10 | 36.71 (20.90-121.87) | 0.025 |
| C-X-C motif chemokine 10 | CXCL10 | 0 | / | / | 11 | 37.93 (21.17-150.89) | 0.028 |
| C-X-C motif chemokine 11 | CXCL11 | 0 | / | / | 8 | 46.63 (21.36-210.40) | 0.025 |

Abbreviations: No., number; SNPs, single nucleotide polymorphisms.

**1.4 Supplementary Table 4. Description of the selected instrumental variables associated with circulating IL-4, IL-24, and CCL19, and their associations with AQP4-ab-positive NMOSD.**

| Cytokines | SNP | Chr:pos | EA | EAF | Cytokine | | | AQP4-ab-positive NMOSD | | |
| --- | --- | --- | --- | --- | --- | --- | --- | --- | --- | --- |
|  |  |  |  |  | Beta | SE | P-value | Beta | SE | P-value |
| IL-4 | rs11989681 | 8:22649912 | A | 0.5982 | 0.0678 | 0.0136 | 6.19E-07 | 0.1804 | 0.142995 | 0.2071 |
|  | rs12641440 | 4:185529634 | C | 0.9065 | -0.1181 | 0.025 | 2.31E-06 | 0.6619 | 0.331699 | 0.04599 |
|  | rs13418255 | 2:107668175 | A | 0.7233 | 0.0701 | 0.0153 | 4.61E-06 | 0.418 | 0.180614 | 0.02065 |
|  | rs2502347 | 1:243071390 | T | 0.2195 | -0.077 | 0.0167 | 4.01E-06 | -0.0098 | 0.163143 | 0.9521 |
|  | rs4876407 | 8:119274249 | T | 0.9451 | -0.1391 | 0.0299 | 3.28E-06 | -0.2684 | 0.31857 | 0.3995 |
| IL-24 | rs167018 | 7:155623090 | A | 0.4443 | 0.0657 | 0.0136 | 1.36E-06 | 0.334 | 0.140182 | 0.01719 |
| CCL19 | rs10242459 | 7:3911280 | A | 0.8219 | 0.0729 | 0.0154 | 2.20E-06 | 0.157 | 0.184267 | 0.3942 |
|  | rs3792790 | 5:150442171 | A | 0.4736 | 0.0551 | 0.012 | 4.40E-06 | 0.2075 | 0.139324 | 0.1364 |
|  | rs4554017 | 4:145124024 | T | 0.3904 | 0.0596 | 0.0122 | 1.03E-06 | -0.0327 | 0.14242 | 0.8184 |
|  | rs62292952 | 3:132200719 | T | 0.8822 | 0.2346 | 0.0185 | 7.53E-37 | 0.4262 | 0.24156 | 0.07767 |
|  | rs6870560 | 5:174187919 | T | 0.8559 | 0.0863 | 0.0169 | 3.28E-07 | -0.0407 | 0.191741 | 0.8319 |
|  | rs73257451 | 8:61418130 | T | 0.989 | -0.3645 | 0.0756 | 1.43E-06 | -1.3022 | 0.558037 | 0.01962 |

Abbreviations: SNP, single-nucleotide polymorphism; Chr, chromosome; pos, position; EA, effect allele; EAF, effect allele frequency; SE, standard error; IL-4, interleukin-4; IL-24, interleukin-24; CCL19, C-C motif chemokine 19.

**References**

1. Zhao, J.H. *et al.* Genetics of circulating inflammatory proteins identifies drivers of immune-mediated disease risk and therapeutic targets. *Nature immunology* **24**, 1540-1551 (2023).

2. Estrada, K. *et al.* A whole-genome sequence study identifies genetic risk factors for neuromyelitis optica. *Nature communications* **9**, 1929 (2018).
